# Contribution of draft cattle to rural livelihoods in a district of southeastern Uganda endemic for bovine parasitic diseases: an economic evaluation

**DOI:** 10.1186/s13071-015-1191-9

**Published:** 2015-11-05

**Authors:** Walter O. Okello, Dennis Muhanguzi, Ewan T. MacLeod, Susan C. Welburn, Charles Waiswa, Alexandra P. Shaw

**Affiliations:** Division of Infection and Pathway Medicine, School of Biomedical Sciences, College of Medicine and Veterinary Medicine, University of Edinburgh, Edinburgh, United Kingdom; Department of Biomolecular and Biolaboratory Sciences, School of Biosecurity, Biotechnical and Laboratory Sciences, College of Veterinary Medicine Animal Resources and Biosecurity, Makerere University, P.O. Box 7062, Kampala, Uganda; Avia-GIS, Risschotlei 33, B-2980 Zoersel, Belgium

**Keywords:** Draft cattle, Animal traction, Trypanosomiasis, Gross margin analysis, Household income, Uganda

## Abstract

**Background:**

A study was conducted in Tororo District in eastern Uganda to assess the socio-economic contribution of draft cattle to rural livelihoods. The aim of the study was to empirically quantify the economic value of draft cattle thus contributing to understanding the impact of endemic parasitic diseases of cattle on livestock productivity and subsequently household income, labor and food security.

**Method:**

A total of 205 draft cattle keeping households (*n* = 205) were randomly selected and structured household questionnaires were administered, focusing on work oxen use, productivity, inputs and outputs. The data obtained was analyzed using standard statistical methods and used to calculate the gross margin from the draft cattle enterprise. Secondary data were obtained from focus group discussions and key informant interviews and these were analyzed using Bayesian methods.

**Results:**

The study showed that, apart from being labor saving, the use of animal traction is highly profitable with the gross margin per year from the use of draft cattle amounting to 245 United States dollars per work oxen owning household. The cash obtained from hiring out draft animals was equivalent to nearly a quarter of the average local household’s monetary receipts. It also revealed that endemic bovine parasitic diseases such as trypanosomiasis and tick-borne diseases reduced draft cattle output by 20.9 % and potential household income from the use of draft oxen by 32.2 %.

**Conclusion:**

The presence of endemic cattle diseases in rural Uganda is adversely affecting the productivity of draft cattle, which in turn affects household income, labor and ultimately food security. This study highlights the contribution of draft cattle to rural livelihoods, thus increasing the expected impact of cost-effective control strategies of endemic production limiting livestock diseases in Uganda.

## Background

Animal traction refers to the use of domestic and partly domesticated animals for the purposes of tillage and transport [1]. The terms animal traction and draft animal power are used interchangeably. Worldwide, animal traction is an important reason for keeping livestock, particularly in poor rural societies, given that it is a cost-effective ‘labor-saving’ technology for small scale and poor subsistence farmers compared to other forms of mechanization and the use of human power [2, 3]. Also, it is envisaged that these trends will continue in the near future as it is expected that agricultural intensification will rise due to increased population pressure and access to markets [4].

Studies have been carried out on animal traction in different parts of Africa, describing both draft output and other benefits. In western Africa, earlier studies comparing draft ox-owning households with non ox-owning ones in Mali revealed that households with work oxen had better crop yields, lower labor requirements and higher incomes depending on the availability of fallow land [5]. More recent studies from Botswana, South Africa and Zimbabwe showed that draft cattle provided the highest proportion of livestock-derived income (up to 75 %) compared to milk, market sales and manure [6]. In eastern Africa, farmers using animal traction were found to have a higher yield and operated at a higher economic efficiency compared to those using hand held hoes [7]. In Ethiopia, better health and higher numbers of work oxen were clearly associated with an increase in cultivated area per household [8].

Despite its obvious benefits, active promotion of animal traction, and current research into the external social and economic drivers of its role in the fluid dynamics of farm operations, is limited [9]. These limited studies, coupled with the long time intervals required to study its impact, are thought to be contributing factors to the under estimation of the livestock sector’s contribution to the gross domestic product hence cyclical under-funding of the sector in developing countries [10, 11]. This situation is made worse by the perception that animal traction is considered “archaic” [7] and is therefore neglected in most agricultural policies [12].

Draft cattle are typically found in the mixed farming systems [13] particularly within the arid and semi-arid tropics and sub-tropics where crop-livestock interactions are common [14]. Also, in these regions and particularly sub-Saharan Africa, farmers face an immense challenge in keeping cattle productive, as a range of endemic parasitic and vector-borne diseases affect their livestock [15]. Thus, it is critical to assess the interaction between such diseases and work output from draft cattle [16, 17].

The main objective of the study was to determine the economic value of draft cattle enterprise using gross margin analysis, thus developing a standard evaluation method for assessment of animal traction in this area. Other objectives were to quantify the cost of endemic production-limiting diseases in cattle, contributing towards the wider research program on the cost-effectiveness of innovative farmer-led technologies to control these diseases [18].

At the farm level, gross margin analysis is normally used to assess the contribution of a particular crop, tree or livestock enterprise to farm income. To date, gross margin analysis has rarely been used for evaluating animal traction, although other methods such as multi-year partial budgeting [19], regression modeling [1, 20], production function modeling [7] and farm modeling [21] have been used in the past. Draft animal power is well captured by gross margin analysis given its dual role as a farm input in the form of traction, whilst simultaneously generating income through the sale or hiring of animals [22]. It also has the advantage of including changes in the herd value, accounting for the effects of changes in herd composition through births and deaths [23].

The study was done in 2012 in Tororo district. The district comprises an area of 1175 km^2^, bordering Kenya in southeastern Uganda. The major economic activity in the district is subsistence mixed farming, with over 80 % of the population deriving their livelihood from agriculture [24]. The most common endemic vector-borne and parasitic bovine infectious diseases in Tororo district are: East Coast fever or theileriosis caused by *Theileria parva* [25]; babesiosis caused by *Babesia bigemina*; anaplasmosis caused by *Anaplasma marginale*; heartwater or cowdriosis caused by *Ehrlichia ruminantium*; gastroenteritis due to *Haemonchus spp*. infection; fascioliasis caused by *Fasciola gigantica* [26]; trypanosomiasis caused by *Trypanosoma vivax*, *Trypanosoma brucei* and *Trypanosoma congolense* [27]. In addition to animal African trypanosomiasis, human African trypanosomiasis (sleeping sickness) caused by *Trypanosoma brucei rhodesiense* is endemic in this district; impacting severely on human health and livelihoods in the region [28, 29]. It has also been reported that cattle restocking programmes from the district among others pose a risk of spreading human African trypanosomiasis to other regions in Uganda [30]. The most common method of controlling vector borne diseases in the study area is intermittent spraying with pyrethroids and amidines [31].

## Methods

The list of all villages, which were considered as clusters, was obtained from the Ministry of Agriculture, Animal husbandry and Fisheries after which two stage cluster sampling [32] was carried out using CSurvey (University of California, Los Angeles, version 2.0) [33] to estimate the sample size. To avoid bias, this involved presenting the list of villages to an independent epidemiologist who assigned each village a number and then randomly chose a subset from the list. A total of 20 villages were thus selected at the first stage and then 10 households were chosen for the second stage with one person per household being eligible as a draft cattle owner. Based on information from the broader research project in Tororo district [25, 27], it was estimated that draft cattle owners were 20 % of the cattle owning population in Tororo district. The desired level of confidence was 95 % with one half of the confidence interval size being 0.09. The variance estimate was estimated using design effect, which was set as low (i.e. 2.0). In total 205 draft cattle keeping households were interviewed (*n* = 205).

The household and livestock productivity structured questionnaires were administered to all participating households gathering information on household characteristics, livestock diseases, wealth indicators and a twelve month recall of herd dynamics. To select the participating households without bias, the spin dial for direction in CSurvey software was used to pin point the random start household [33]. Only households with draft cattle were interviewed. Focus group discussion and key informant interviews were concurrently utilized to complement the quantitative data [34]. A total of 18 focus group discussions and 28 key informant interviews were carried out on these themes. Apart from Akadot and Rukuli villages, which were not interviewed due to financial constraints, focus group discussions were conducted by selecting and having a discussion with a mixed group of cattle keepers, non cattle keepers, employed herdsmen, animal health technicians, women and business operators within each village. An average of 10.4 persons, with a standard deviation (SD) of 3.9, participated in the focus group discussions which were conducted in the evenings. The key informant interviews were conducted with the elected village chiefs (comprising the Local Council Level One), local veterinary staff and the heads of various women groups. All secondary data obtained from focus group discussions and key informant interviews were selected for stochastic modeling [35]. Uncertainty was modeled with a uniform distribution using upper and lower limits of the data obtained; and the use of Monte-Carlo simulation with 10 000 iterations [36]. This enabled uncertainty to be incorporated in the final point estimate by adding a 95 % credibility interval [35]. Statistical information obtained from the structured questionnaires was expressed as averages (mean) and their standard deviation was cited. The currency used in the data collection was Ugandan shillings, which are converted to United States Dollars (USD) at the exchange applicable at the time of the study (1 USD = 2325 Ugandan shillings) [37].In order to use gross margin analysis to determine the overall contribution of draft cattle to household income, thus allowing for extrapolation to the district level, the draft cattle enterprise was analyzed using the following framework:

$$ \mathbf{GROSS}\ \mathbf{MARGIN}=\left[\mathbf{LIVESTOCK}\ \mathbf{OUTPUT}\right]-\left[\mathbf{VARIABLE}\ \mathbf{COSTS}\right] $$

Where:

i) Livestock output = (draft animals and products sold/consumed/transferred/gifted ‘out’) - (draft cattle brought ‘in’ as purchases/transfers/gifts received) + (increase in herd value over 12 months);
ii) Variable costs = cost of items used exclusively for draft cattle production and for which the quantity used varies in the short-term and in relation to the quantity of output.

According to the focus group discussions, the local price of labor paid during times of peak agricultural activity was USD 1.6 per day on average, with a credibility interval (CI) of 1.2–2; accordingly the value of family labor associated with draft cattle plowing was conservatively estimated to be 30 % of this or USD 0.48 [38]. Conventionally, in farm budgets casual labor is considered a variable cost whereas family labor is considered at the fixed cost level. However, the use of animal traction is crucially bound up with changing labor requirements. It involves both labor cost specifically for managing draft animals and an overall labor saving due to their use. Accordingly, these varying labor components were, respectively, included in the variable costs and in livestock output, where the value of own-farm use of animal traction was based on the equivalent hand plowing labor saved. For the economic analysis, the household data was pooled, and then averages (mean) per household and per draft male calculated. The software R (R development core team, version 3.2.1) was used for the statistical analyses [39].

### Ethical approval

This study was among a set of other related studies reviewed by the Makerere University College of Veterinary Medicine Animal Resources and Biosecurity ethical review board for compliance to Animal use and Care Standards. It was then forwarded to the Uganda National Council for Science and Technology and approved under approval number HS1336.

## Results

The average number of household members was 7.2 (SD 3.6), of whom 2.6 were adults over 18 years old and 4.6 children, with 97 % of households being male-headed. The main occupation was agriculture: 93.4 % of draft cattle keepers relied solely on crop and livestock farming, with only 6.8 % deriving additional non-farm income (such as running local businesses or receiving cash from relatives who reside outside the district) . The average area cultivated per household was 3.6 acres (SD 1.7). Eighty three per cent of the farmers interviewed owned their own ox-plow, while none owned or used a tractor for plowing.

The total number of cattle owned by those participating in the study was 1003, with each household owning an average of 4.9 cattle; 0.3 sheep, 2.3 goats, 1.5 pigs, 11.6 chickens and 1.4 other livestock. Out of the 1003 cattle, there were 280 (27.9 %) work oxen (a term which refers to both bulls and castrated bulls in the study area, 270 (26.9 %) adult females, 172 (17.1 %) heifers, 158 (15.8 %) young males, 69 male calves (6.9 %) and 54 female calves (5.4 %) as shown in Table 1. Among the calves in the herd there are thus 1.2 males to every female, reflecting the importance of draft power, with farmers indicating during focus group discussions that they give preferential care to male calves for this reason. Virtually all adult male cattle (bulls and oxen) were used for draft and young males were trained for the same purpose. No female cows were used for plowing.

**Table 1:** Herd structure of cattle among draft cattle keepers in Tororo district

Age category	Cattle herd composition (%, *n* = 1003)
Work oxen	27.9
Cows	26.9
Heifers	17.1
Young males	15.8
Male calves	6.8
Female calves	5.5

The total number of draft cattle was 438, representing 43.7 % of the sample households’ cattle herd, with an average of 2.1 (SD 0.6) draft cattle owned per household as given in Table 2. This mean is very representative, since of the 205 households interviewed, 151 households had 2 draft cattle. Of the remainder, 20 households had only 1 draft bovine, 22 households had 3 draft cattle, 11 households had 4 draft cattle and 1 household had 6 draft cattle (Table 2). Since nearly 75 % of all households have the same herd size (2 oxen) it was felt that analyzing the pooled data from the whole sample would provide representative results for the economic analysis.

**Table 2:** Summary of annual pooled data on days worked by draft oxen per household

Number of work oxen owned per household	Number of households interviewed	Percentage of interviewed households	Average days worked per work oxen	Number of days plowing on own farm	Number of days hired out for plowing on other people’s farms	Percentage of plowing days spent on own farm
1	20	9.8	32.9	21.1	11.8	64.1
2	151	73.7	53.4	16.0	37.4	30.0
3	22	10.7	53.1	14.5	38.6	27.3
4	11	5.4	53.5	12.2	41.3	22.8
4+	1	0.5	52.0	10.0	42.0	19.2
Whole sample	205	100.0	51.6	16.1	35.3	31.2

At the start of the year leading up to this study, the total number of cattle comprised 1 044 of which 349 were adult males and 136 young males. During that year 117 cattle died: 27 adult males, 8 young males, 24 cows, 21 heifers and 37 calves. Thus the herd mortality rate was 11.2 % with draft cattle representing 29.9 % of the total deaths. The reasons for cattle deaths were diseases, mentioned by 96.4 % of the respondents, road accidents, mentioned by 2.2 % of the respondents and 1.4 % unknown. Data obtained from the structured questionnaire showed that adult draft cattle were valued at USD 427 (SD 48.9) and young male draft cattle at USD 185 (SD 96.3).

All the questionnaire respondents used draft cattle both for plowing their own farms, and hired them out during the two planting seasons. Secondary uses for draft cattle included other heavy manual labor, such as pulling logs. No farmers used their draft cattle for weeding or seeding. Also, the structured questionnaires revealed that on average draft cattle start work at 2.6 years (SD 0.6) and finish at 11.1 years (SD 2.4), resulting in a working life of 8.5 years. Within this time however, farmers indicated a time investment of an average of 18 months (CI: 12.2–23.7) for training, so according to information obtained from the focus group discussions, optimum efficiency was generally not reached until the animal was 4.1 years of age, which tallies with the information obtained from the questionnaires. Analysis of the data obtained from the structured questionnaires indicated that, working a mean 4.3 h a day (SD 0.4), it takes a pair of draft cattle 2.2 days (SD 0.7) to plow 1 acre of land, equivalent to a working rate of 0.5 acres per day. The average number of annual work days was 51.6 (SD 6.8), consisting of 16.1 days (SD 2.5) on the farmer’s own land, 35.3 days (SD 8.2) hired out on other farms, and 0.2 days (SD 0.4) doing other work. Also, the only group whose animals worked mainly on their own farm (64.1 % of days) were the households with only one ox. All households with one work oxen ultimately hired or borrowed extra ones from other farmers. Data on the number of days worked by draft oxen in each household is summarized in Table 2.

All farmers accompanied their draft cattle when they hired them out. The total annual human labor requirement for plowing using draft cattle was calculated as equivalent to 102.8 days, based on 2 people spending an average of 16.1 days annually plowing their own land, and 35.3 days plowing land belonging to others (Table 2). Cash payments for hiring of draft cattle services occurred in 92 % of cases, with the remaining 8 % receiving in-kind payments such as chicken or maize. Farmers charged a daily average rate of USD 8.6 (SD 4.1) for plowing, and USD 4.3 (SD 6.4) for any other draft work. Using focus group discussions and key informant interviews, a comparison was also done between the use of draft cattle and the two main alternatives to draft; hand held hoes and tractors. It was found that it would take 2 people, plowing 3.4 (CI: 2–4.9) hours a day, 12 (CI: 10–13.9) days to plow 1 acre of land using hand held hoes. This is equivalent to a work output of 0.1 acres per day. Thus, the total annual labor that farmers require to plow their own farm using a hand held hoe was estimated at 172.8 labor days (based on 2 people taking 12 days per acre, 2 planting seasons and an average farm size of 3.6 acres). In comparison, it was revealed that a tractor can plow 5.4 (CI: 3.1–7.8) acres a day. Also, data obtained from focus group discussions showed that the hiring rate for a tractor was USD 36.3 (CI: 26.5–46.8) per acre. Table 3 provides a summary of the work output of draft cattle from the analyzed data obtained from the structured questionnaire. The SD for the means is included in the table. Lastly, Table 4 summarizes all the results attained from the focus group discussions and key informant interviews; showing the values and the CI.

**Table 3:** Draft cattle work output in Tororo district

Output Parameter	Duration	Standard deviation (SD)
Age at which work oxen start work	2.6	0.6
Age at which working life ends	11.1	2.4
Time for a pair of draft cattle to plow one acre of land (days)	2.2	0.7
Hours worked per day	4.3	0.4
Days worked plowing own farm per year	16.1	2.5
Days worked plowing other people’s farm per year	35.3	8.2
Days worked doing other draft work per year	0.2	0.4
Total days worked per year	51.6	6.8

**Table 4:** Summary of all results obtained from secondary data sources

Parameter	Value (95 % CI)
Price of local labor at peak agricultural activity (in USD)	1.6 (1.2–2.0)
Mean rural income per person per month in Tororo district (in USD)	38.8 (30.4–47.3)
Period taken by 2 people to plow 1 acre of land (in days)	12 (10–13.9)
Period worked by 2 people per day to plow 1 acre of land using hand held hoe (in hours)	3 (2–3.9)
Duration of time taken to train young male cattle for work (in months)	18 (12.2–23.7)
Size of land plowed by a tractor per day (in acres)	5.4 (3.1–7.8)
Hiring rate of tractor per acre (in USD)	36.3 (26.5–46.8)

Economic analysis of the questionnaires revealed that the total combined annual income (both cash and in kind) from all households from hiring out draft cattle was USD 60 935. An additional benefit, in the form of an averted cost is the cost of human labor that would be used to plow by hand if a farmer did not own his own draft cattle, valued at USD 19 997 across 205 households per year.

One year recall showed 40 adult draft cattle across the 205 households were sold during the past year at a total value of USD 17 101. Four draft cattle were “gifted” to others as loan repayments, at a total value of USD 1 710, whilst two were stolen, worth USD 855. Paradoxically, whilst for the cattle owner thefts clearly represent a loss, from the societal point of view they are an output from the livestock farm. The total value of draft output and draft cattle offtake was therefore USD 100 598. The total number of draft cattle bought in one year by the 205 households was 30, at a total value of USD 5 561. A further four young animals were received as gifts into the herd, valued at USD 1 710, resulting in a total value of draft cattle into the herd of USD 7 271. At the start of the 12 month period, the total opening draft cattle valuation was USD 174 417 and at the end of the year the closing valuation was USD 148 966. Thus, the herd value (closing valuation minus opening valuation) was negative USD −25 421. Sensitivity analysis was done to find out the effect of death on the closing herd value and the change in herd value for a ‘without death’ scenario was just positive, coming to USD 628.6. It is noteworthy that draft animals at the end of their working life were always sold for money, with no informal transfers out of the herd or home slaughter taking place.

The variable costs consisted first, of the cost of keeping the draft animals (animal health inputs, vector control, feed, ropes, fines for crop damage), second, of occasional costs for replacing sick or absent draft animals and third, of the value of the farmers’ labor expended on draft work, either on the household’s farm or when accompanying draft animals that were hired out. Trypanosomiasis was perceived to be the most common disease affecting cattle in the district, mentioned by 98.2 % of the farmers. East Coast fever was mentioned by 36.8 % of the respondents. Households indicated an average of 13.6 days (SD 8.1) lost to work per year as a result of diseased cattle, resulting in a total 2 788 days of lost work over the 205 households. The total variable cost for all the sampled households was USD 17 522. Given the total livestock output was USD 67 905 and the total variable cost as USD 17 517, then the total gross margin for all the households was USD 50 383 as shown in Table 4.

The income (cash and income in kind excluding value of home labor saved) received for hiring draft oxen for plowing per household was USD 297 while that from other components of draft oxen output was USD 34 per household giving a total livestock output per household of USD 331. The average variable costs incurred per household came to USD 86. By subtracting variable costs from livestock output, then average gross margin for each draft cattle enterprise comes to USD 245 per year as shown in Table 5.

**Table 5:** Draft cattle enterprise gross margin calculation for the whole study sample

Item	Value (USD)
a) Total value of livestock products, animals sold or transferred out of the herd	
● Annual income from hiring out draft cattle	60 935
● Animals sold	17 101
● Animals given out as loan repayments	1 710
● Value of animals stolen	855
● Value of human labor saved	19 997
Subtotal (a)	100 598
b) Total value of animals brought in to the herd	7 271
c) Change in herd value during the year	−25 421
d) Total livestock output (a-b + c)	67 905
e) Total variable cost	−17 522
f) Total gross margin (d-e)	50 383

The net cash income received per household using draft cattle was also calculated. The total cash received (excluding income in kind and home labor cost averted) for hiring out draft oxen for plowing was USD 278, other draft work USD1 and sale of draft oxen was USD 83 per household totaling USD 362. However, each household used USD 27 to purchase draft oxen and incurred monetary variable costs of USD 36 thus the net cash income per household was USD 299 as shown in Table 7.

A sensitivity analysis on labor cost was done to ascertain its effect on the total and household gross margin. Using 50 % of the labor cost (USD 0.8) on all calculations; the total gross margin of all households was USD 56 917 or USD 278 per household and the contribution of draft power to the total livestock output per household was 75.5 % while using 100 % of the labor cost it was USD 80 575, USD 393 and 53.2 % respectively.

## Discussion

The herd composition revealed that draft cattle represented 43.7 % of the cattle owned by draft cattle keepers and that 49.2 % of cattle kept were male. This contrasts with the composition of herds kept by farmers or pastoralists focusing on milk production and herd growth, where females usually account for over two thirds of the herd, for example 70 % in Maasai herds with 60 % of the herd consisting of cows and weaned heifers [40]. The herd structure found in Tororo corroborates other information from Africa, such as: Zimbabwe where draft cattle represented 40 % of the total cattle herd in communal areas [21], southern Mozambique where 25 % of the herd are work oxen [41] and Serere district (Uganda) where 36 % of the cattle were used for draft [42].

Overall, the benefits of using draft cattle greatly exceeded the cost of keeping them; the total gross margin for each household due to draft cattle enterprises was positive (USD 245), indicating their use was highly profitable. Studies done in Botswana [43], South Africa [44] and northern Ghana [45] also found this to be the case. In Tororo, the study revealed that draft oxen add substantially to farmers’ cash income, largely through cash payments received from hiring them out, since the average net cash income from hiring of USD 299 per household, was actually higher than the draft cattle enterprise gross margin of USD 245. This was because the value of negative non-cash items in the gross margin calculation (such as reduced herd value, animals transferred into the herd and variable costs) was greater than that of the positive non-cash items (such as payments in kind for hiring draft oxen, home labor saved and animals transferred out of the herd) as shown in Tables 6 and 7. These values obtained are comparable to those estimated for South Africa [46] where own farm plowing was valued at USD 177, lending animals out for plowing at USD 11 and hiring them out at USD 29 per household.

**Table 6:** Draft cattle enterprise gross margin calculation per household

Item description	Value (USD)
• Income from draft oxen power (including the value of home labor saved)	297
• Other components of draft oxen output	34
Total livestock output	331
• Variable costs	86
Total gross margin from using draft oxen	245

**Table 7:** Draft cattle enterprise net cash income calculation per household

Item description	Value (USD)
• Cash received from hiring out draft cattle for plowing and other draft work	279
• Cash received from sale of draft oxen	83
Subtotal	362
• Draft oxen bought	27
• Variable costs	36
Subtotal	63
Net cash received per household	299

The average annual cash earned per adult in the study area is estimated at USD 465 [47]. Considering the average number of adults per household in the study was 2.6, the average annual monetary receipts per household would be USD 1 209. The monetary receipts from using draft oxen came to USD 299 per draft-animal keeping household (Table 7), equivalent to 24.7 % of this figure.

Given income (both cash and in kind) received from the use of draft cattle for each household was USD 297 and from other components of output was USD 34, then the income from hiring out draft power represented 89.7 % of the total livestock output per household. Therefore the main benefit of using draft oxen in Tororo district was income generated from draft power. It should be noted that farmers owning 2 or more draft cattle contributed most of the pooled income gained from draft cattle hire as they compose 88.2 % of draft cattle keepers in Tororo district.

A person with no draft cattle has three alternatives; a) to hire in draft oxen from another farmer, b) use manual labor (a hand held hoe) or c) use a tractor. To hire a pair of oxen costs a non-draft keeper USD 19 per acre. Hiring two manual laborers would cost USD 14 per acre and hiring a tractor would cost USD 36 per acre. Use of draft cattle for plowing is therefore less costly than tractors, but more costly than use of a hand held hoe, however, manual labor has the lowest daily work output, thus being a more expensive option in the long term. More importantly, in rural areas such as Tororo district, the majority of farmers rely on rain-fed agriculture, thus timely preparation of land for crop planting is critical to ensure a high crop yield. Lack of animal traction can therefore lead to planting delays or even to land being left fallow; as demonstrated by studies in Zimbabwe that estimated planting delays could lead to yield losses of 1–3 % per day [48]. Draft cattle use also offsets the drudgery associated with plowing with human power using a hand held hoe. Thus, ownership of draft cattle meant that the total labor requirement for their own farm was only 32.2 days per household, compared to the 172.8 labor days that would have been required if they used manual labor, freeing up substantial time for farmers to do other work. The results from the current study therefore demonstrate animal traction to be an efficient means of land preparation, contributing to improved food security by reducing the likelihood of crop failure.

The 3.4 % annual mortality rate of draft cattle was perceived by farmers to be almost entirely due to disease, resulting in a reduced herd value that ultimately affected the gross margin. Work days lost due to draft cattle illness was calculated at 13.6 days per year per household, which when added to the current number of days worked per year (51.6) equates to a potential of 65.2 days per year that could be worked if cattle did not become sick. Therefore across 205 households, the total lost income as a result of livestock disease was USD 23 898, equivalent to a decrease of 32.2 % of the potential total gross margin from use of draft oxen per household. The reduced work capacity as a result of livestock disease in this study was 20.9 %, in addition to the aforementioned loss of household income, this severely impacts the wider community through lack of available animals for plowing other people’s farms, extra human effort required to hand plow, and potentially reduced crop yields as a result of delays in plowing [48] with an ultimate effect on overall food security.

Overall, use of gross margin analysis proved an effective methodology for assessment of the economic contribution of draft animal power at the household level in terms of income generation and labor saving. However, one of the challenges of using gross margin analysis in this way is extrapolating findings to the macro-economic level in order to achieve policy impact. This is particularly important in that, ultimately animal traction is not an end product but an input into crop output, hence not directly reflected in national accounts or most livestock models. Therefore, the authors recommend complementing it with other economic models and accounting frameworks such as Food and Agriculture Organization of the United Nation’s (FAO) System of Economic Accounts for Food and Agriculture (SEAFA) [49]. At the micro-economic level there is still farm level research work to be done on quantifying the impact of animal traction on crop yields and on the size of the cropped area per household and hence the overall impact of endemic bovine diseases.

## Conclusions

This research is among the few studies in recent years in Africa, and the first evidence from Uganda, to quantify the socioeconomic impact of using animal traction in a mixed crop-livestock production system in which bovine parasitic diseases are endemic. It indicates animal traction to be a highly profitable enterprise at the household level, not just in terms of its contribution to crop production but also its various roles in livelihoods sustainability such as household income generation, cattle sales and social capital. However, endemic production-limiting parasitic diseases such as trypanosomiasis and tick-borne diseases reduce draft oxen work output and household income; increasing farmers’ vulnerability to economic and food insecurity. Therefore, the need for appropriate systemic investments in animal health service delivery and the promotion of animal traction as a means of improving rural livelihoods is an important recommendation to come out of this research.

## Abbreviations

CI: credibility interval (95 %)
SD: standard deviation
USD: United States Dollar
